# The potential role of gut microbiota-derived metabolites as regulators of metabolic syndrome-associated mitochondrial and endolysosomal dysfunction in Alzheimer’s disease

**DOI:** 10.1038/s12276-024-01282-3

**Published:** 2024-08-01

**Authors:** Young Hyun Jung, Chang Woo Chae, Ho Jae Han

**Affiliations:** 1 Department of Physiology, College of Medicine, Soonchunhyang University, Cheonan, 31151 Korea; 2 Department of Veterinary Physiology, College of Veterinary Medicine, Research Institute for Veterinary Science, and BK21 FOUR Future Veterinary Medicine Leading Education & Research Center, Seoul National University, Seoul, South Korea

**Keywords:** Mitochondria, Endosomes, Molecular neuroscience

## Abstract

Although the role of gut microbiota (GMB)-derived metabolites in mitochondrial and endolysosomal dysfunction in Alzheimer’s disease (AD) under metabolic syndrome remains unclear, deciphering these host–metabolite interactions represents a major public health challenge. Dysfunction of mitochondria and endolysosomal networks (ELNs) plays a crucial role in metabolic syndrome and can exacerbate AD progression, highlighting the need to study their reciprocal regulation for a better understanding of how AD is linked to metabolic syndrome. Concurrently, metabolic disorders are associated with alterations in the composition of the GMB. Recent evidence suggests that changes in the composition of the GMB and its metabolites may be involved in AD pathology. This review highlights the mechanisms of metabolic syndrome-mediated AD development, focusing on the interconnected roles of mitochondrial dysfunction, ELN abnormalities, and changes in the GMB and its metabolites. We also discuss the pathophysiological role of GMB-derived metabolites, including amino acids, fatty acids, other metabolites, and extracellular vesicles, in mediating their effects on mitochondrial and ELN dysfunction. Finally, this review proposes therapeutic strategies for AD by directly modulating mitochondrial and ELN functions through targeting GMB metabolites under metabolic syndrome.

## Role of gut microbiota metabolites in metabolic syndrome-associated Alzheimer’s disease

Although mitochondrial and endolysosomal network (ELN) impairment in metabolic syndrome is considered a risk factor for neurodegenerative diseases, the regulatory role of gut microbiota (GMB)-derived metabolites in this dysfunction remains unclear. This research explores the roles and molecular mechanisms of mitochondrial dysfunction, ELN abnormalities, dysregulation of mitochondria-ELN crosstalk, and changes in GMB and its metabolites in metabolic syndrome, especially in relation to Alzheimer’s disease (AD). The researchers conclude by highlighting the potential of targeting GMB and its metabolites to develop new AD treatments, especially for those with metabolic syndrome. They suggest that understanding and modulating the links between gut health, mitochondrial function, and ELN activity could lead to new management strategies for AD in the context of gut microbiota and its metabolites.

This summary was initially drafted using artificial intelligence, then revised and fact-checked by the author.

## Introduction

Alzheimer’s disease (AD), a leading cause of dementia worldwide, currently affects approximately 416 million individuals and poses a significant concern to global health care systems^1^. AD is primarily associated with a progressive decline in cognitive ability, accompanied by amyloid beta (Aβ) plaques and tau tangles; these two pathologies are the hallmarks of its pathogenesis^2^. These features underlie neuronal damage and loss, indicating that these conditions are devastating. Although the etiology of AD is not fully understood, it is believed to be a multifactorial disease affected by genetic, environmental, and molecular factors^2–4^. Furthermore, increasing acknowledgment of the cellular disruptions in AD pathogenesis highlights the complexity of this condition, expanding beyond the traditional focus on Aβ plaques and tau tangles to encompass a wider range of pathological factors.

A broader understanding combined with epidemiological studies revealed that metabolic syndrome, a condition characterized by hyperglycemia, insulin resistance, and dyslipidemia, which increases the risk of diabetes mellitus (DM) and obesity, leads to oxidative stress-mediated neuronal death and exacerbates the accumulation of Aβ and tau tangles^5^. Mitochondrial dysregulation induced by imbalanced mitochondrial biogenesis, fusion/fission, mitophagy, a specialized form of autophagy targeting mitochondria, and endolysosomal network (ELN) abnormalities are common pathophysiological mechanisms of AD and metabolic syndrome^6–9^. In particular, the functions of the mitochondria and the ELN are coordinated with each other in response to cellular metabolism and related signaling. In mitochondrial dysfunction, the cell inhibits nutrient-sensing AMP-dependent protein kinase (AMPK) signaling, which reduces lysosomal activity, resulting in mitochondrial damage and dysfunction. Reciprocally, the cell responds to lysosomal deficiency by inhibiting the generation of new mitochondria, which appears to restrict lysosomal function. This condition also leads to mitochondrial impairment and lysosomal damage^10^. Although the precise molecular mechanisms involved require further investigation, these findings indicate that mitochondrial and ELN dysfunction are significant factors in the pathogenesis of AD associated with metabolic syndrome. Furthermore, because neurons have two morphologically distinct polarized compartments, the supply of energy by mitochondria and the transport and removal of vesicles/organelles by the ELN are critical for maintaining cell function. Therefore, further studies are needed to investigate the intricate connections between mitochondrial and ELN dysfunction and AD pathogenesis to develop disease treatment strategies.

Since dysregulation of the gut–brain axis (GBA) has been reported in AD, elucidating microbiota dysbiosis is important for understanding disease pathogenesis^11,12^. Gut dysbiosis is also observed in metabolic syndromes, suggesting that changes in the gut microbiota (GMB) and its metabolites may play a role in the development of metabolic syndrome-associated AD^13^. Transplantation of the fecal microbiota in patients with metabolic syndromes has emerged as a treatment strategy for AD that focuses primarily on modulating GMB^12^. Furthermore, the administration of gut-derived metabolites, such as butyrate, improves the brain’s energy metabolism and neurotransmitter synthesis by modulating oxidative stress and autophagy, a cellular process that removes dysfunctional components through lysosome-mediated degradation^14^. Although some metabolites enhance mitochondrial function and aid in the removal of harmful proteins^14^, others can increase oxidative stress and impede autophagy, exacerbating Aβ and tau pathologies^15^. Therefore, understanding the alterations and effects of the microbiota and its metabolites in metabolic syndrome is essential for elucidating AD regulatory mechanisms. In this review, we aim to provide a comprehensive overview of the interconnected roles of mitochondrial dysfunction, ELN abnormalities, and changes in GMB and its metabolites in the pathogenesis of AD associated with metabolic syndrome. By exploring the mechanisms through which GMB-derived metabolites influence these cellular processes, we hope to shed light on novel therapeutic targets and strategies for managing AD, especially in the context of metabolic syndrome. We first discussed the pathogenesis of AD associated with metabolic syndrome, focusing on mitochondrial and ELN dysfunction. Next, we explored alterations in the GMB composition and metabolites in metabolic diseases and their potential impact on AD development. Finally, we highlighted the therapeutic implications of targeting the GMB–metabolite–mitochondria–ELN axis in AD and propose future research directions to address current knowledge gaps.

## Pathogenesis of AD associated with metabolic syndrome

**Fig. 1:**
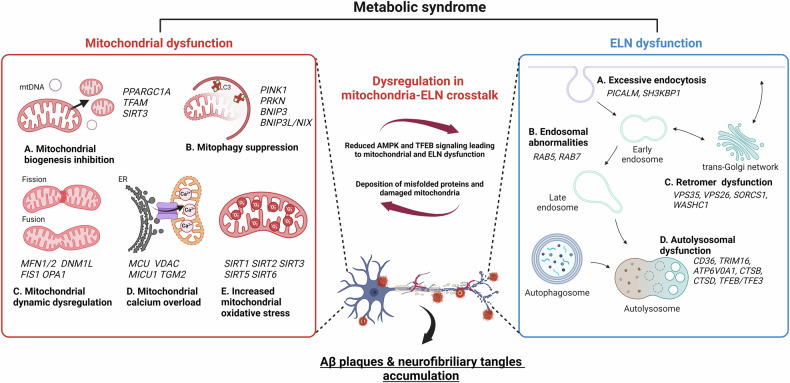
Involvement of mitochondrial and ELN dysfunction in metabolic syndrome-associated Aβ plaques and the accumulation of neurofibrillary tangles. AD progresses concurrently with mitochondrial dysfunction (left) and ELN dysfunction (right). This results in the deposition of misfolded proteins and the accumulation of damaged mitochondria. Both the AMPK and TFEB signaling pathways are suppressed by each other. The occurrence of metabolic syndrome may be associated with dysfunctions related to mitochondrial biogenesis, mitophagy, fusion and fission dynamics, calcium homeostasis, and antioxidative mechanisms. The expression of related genes is influenced by metabolic cues. Additionally, there are issues related to the process of endocytosis, the formation of endosomes, the retromer complex, and the function of autolysosomes. As a result, metabolic syndrome significantly contributes to the crosstalk between mitochondria and the ELN. Inefficient ATP production, impaired mitochondrial homeostasis, and disrupted autophagy in neuronal cells lead to the accumulation of misfolded proteins and damaged organelles. Overall, metabolic syndrome induces the formation of Aβ plaques and the accumulation of neurofibrillary tangles. Created with BioRender.com.

Epidemiological research has shown that metabolic diseases such as DM and obesity increase the risk of developing AD^5,16^. Patients with type 2 diabetes have an additional 35% greater chance of developing AD if they have mutations that are strong genetic risk factors for AD^5^. A recent cross-sectional study suggested that the most prominent modifiable risk factor for AD is obesity^16^. These studies have led to further research on how metabolic disease significantly increases the risk of AD. The potential mechanisms underlying this connection in diabetes are hyperglycemia and advanced glycation end products (AGEs)-induced neuronal toxicity, oxidative stress, and insulin resistance in the brain^17^. Furthermore, dyslipidemia in obesity induces neuroinflammation compatible with AD, compromises the integrity of the blood–brain barrier (BBB), and increases the deposition of Aβ plaques and neurofibrillary tangles^17^. We demonstrated that elevated glucose reorganized lipid rafts and increased the BACE1-mediated production of Aβ in neuronal cells^18^. A high level of palmitic acid enhances Aβ production through GPR40-mediated signaling pathways in neuronal cells^12^. These findings highlight the intricate link between metabolic syndrome and the molecular pathogenesis of Aβ production, emphasizing the importance of maintaining metabolic homeostasis to prevent amyloidogenic pathways. In addition, recent evidence has suggested that GMB-derived metabolites are novel factors that strongly interact with neurons, affecting their function and behavior in metabolic diseases. Moreover, substantial evidence suggests that mitochondrial and ELN dysfunctions likely play a crucial role in the pathogenesis of metabolic syndrome-associated AD^6–9^. The following sections discuss the pathophysiological mechanisms of AD pathogenesis, focusing on mitochondrial dysfunction, ELN, and their crosstalk in the context of metabolic syndrome (Fig. 1).

### Mitochondrial dysfunction

Numerous neural processes require significant energy expenditure, with mitochondria serving as the primary energy supplier by generating ATP through oxidative phosphorylation to maintain neuronal homeostasis and functionality. Mitochondria play a crucial role in the production of iron–sulfur centers and heme within neurons, which are integral for the synthesis of presynaptic transmitters in synapses^19^. Given this crucial role, disruptions in mitochondrial function are intricately linked to the mechanisms involved in neurodegenerative diseases, including AD. To date, numerous studies have demonstrated significant mitochondrial abnormalities in the brains of AD patients^8^. This is consistent with the finding that compromised energy metabolism consistently occurs before the clinical manifestation of AD. Consequently, mitochondrial dysfunction has been identified as an early and significant characteristic of AD, indicating its crucial involvement in its development. Since mitochondrial dysfunction is also responsible for the development of metabolic diseases such as diabetes and obesity^20^, elucidating the detailed mechanisms that link mitochondrial dysfunction to AD and metabolic disease is essential.

These mechanisms primarily involve the deterioration of mitochondrial structure and function, including mitochondrial biogenesis and dynamics, mitophagy, interactions between the endoplasmic reticulum (ER) and mitochondria, and the mitochondrial antioxidant system^6,19^. Genetic dysregulation of *PPARGC1A*, *TFAM*, and *SIRT3* in mitochondrial biogenesis; *DNM1L*, *FIS1*, *OPA1*, and *MFN2* in dynamics; *PINK1* and *PRKN* in mitophagy; *MCU*, *TGM2*, and *MICU1* in ER–mitochondrial contact; and *SIRT1, 2, 3, 5*, and *6* in the mitochondrial antioxidant system are strongly associated with the pathogenesis of diabetes^6^. Furthermore, obesity is characterized by impaired mitophagy due to *PRKN* and *BNIP3* downregulation and excessive fission due to *OPA1*, *MFN1*, and *2* dysregulation^21,22^. These findings suggest that specific changes in mitochondrial regulators are important clues when considering the molecular mechanisms by which metabolic syndrome intensifies and exacerbates AD pathology. We previously demonstrated that metabolic syndrome-mediated mitochondrial dysfunction causes Aβ accumulation, neuronal dysfunction, and cognitive impairment. In fact, metabolic stress induces neuronal amyloidogenesis and cognitive impairment through the dysregulation of PINK1-mediated or NIX-mediated mitophagy^14,23,24^, Drp1-mediated mitochondrial fission^25^, TGM2-dependent ER–mitochondria contacts and calcium overload^26^. These findings underscore the importance of metabolic syndrome-mediated mitochondrial dysfunction in AD development, suggesting that factors regulating mitochondrial biogenesis, mitophagy, dynamics, ER–mitochondrial contacts, and the mitochondrial antioxidant system are crucial in understanding the link between metabolic syndrome and AD.

### Endolysosomal dysfunction

The ELN comprises intracellular membranous organelles that undergo dynamic interconversion. Organelles in the ELN include early endosomes, recycling endosomes, late endosomes, autophagosomes, and lysosomes. As neurons possess two different polarized regions and relatively long axons, the essential role of ELNs in facilitating the transportation and elimination of vesicles and organelles is crucial for the preservation of cell functionality. Therefore, ELN dysfunction can lead to a cascade of neurodegeneration due to the accumulation of toxic proteins and damaged organelles. Recent studies have conducted a comprehensive genetic analysis of 891 autophagic and endolysosomal genes in late-onset AD, highlighting that genetic variations in these pathways could contribute to the risk of developing AD^9^. Furthermore, the regulatory mechanisms of endosomal abnormalities and lysosomal impairment in the pathogenesis of AD are endolysosomal protein-mediated abnormal processing mediated by endolysosomal proteins of amyloid precursor protein (APP) and impaired degradation of Aβ and hyperphosphorylated tau^27,28^. Concurrently, since ELNs are essential for the regulation of metabolism through the classification and distribution of signaling receptors and membrane transporters of hormones or metabolites such as the insulin/glucagon receptor, glucose transporter 4, glucagon-like peptide-1 receptor, and low-density lipoprotein receptor^7^, ELN dysfunction is considered an important mechanism in the pathogenesis of metabolic diseases. In particular, the maintenance of proper endocytosis could mitigate pathophysiological changes in diabetic mice^29,30^.

Deficiency in retromer, a protein complex that plays a role in the retrograde transport of cargo from endosomes to the trans-Golgi network, or in the recycling cargo from endosomes back to the cell surface, exhibited abnormal glycemic control^31^. Disruption of lysosomal function and subsequent impairment of autophagic flux have been observed in both diabetes and obesity^29,32^. Therefore, ELN dysfunction is a potential pathophysiological hub between metabolic disease and AD. Our previous studies provide valuable information on ELN-associated mechanisms by which neuronal Aβ and hyperphosphorylated tau accumulate under high glucose conditions through the modulation of PICLAM-mediated APP endocytosis, VPS26a-mediated processing of APP and lysosomal hydrolases, TRIM-mediated lysophagy, and selective autophagy targeting lysosomes^29,31,33^. Although these studies collectively highlight the involvement of ELN dysfunction in AD pathogenesis, more research is needed to investigate other ELN regulators and their contributions to disease pathogenesis. This could help identify potential therapeutic targets for AD associated with metabolic syndrome.

### Dysregulation of mitochondrial-ELN crosstalk

Mitochondrial dysfunction impacts components of the ELN, such as early (returning receptors on the cell surface) and late (directing cargo toward lysosomal degradation) endosomes^34^. A recent study demonstrated that endosome–mitochondria associations through phosphoinositide 3-kinase (PI3K)–voltage-dependent anion channel 2 (VDAC2) interactions modulate endosomal maturation^35^. Early endosomes also regulate mitochondrial function through Rab5 GTPase and its adaptor protein-mediated mitophagy^36^. Although the molecular mechanisms underlying the formation and regulation of mitochondria-endosome contact sites are still largely unknown, the evidence suggests that physical contacts between the two organelles allow for the direct transfer of essential molecules, such as iron and cholesterol, and that mitochondrial impairment may disrupt the energy balance and cellular signaling pathways necessary for proper endosomal function^37^. Given the limited literature on the direct interactions between mitochondria and early/late endosomes, further research is essential to investigate the molecular pathways connecting mitochondrial dysfunction, endosomal abnormalities, and metabolic perturbations in the context of AD pathogenesis. Similar to mitochondria–endosome interactions, the contact between mitochondria and lysosomes is also regulated by Rab7 GTPase in a bidirectional manner, and this regulation occurs through the modulation of both mitochondrial and lysosomal dynamics, as well as the transfer of metabolites between these two organelles, and disruptions in these contact sites have been associated with AD^38,39^. Furthermore, it should also be noted that dysfunction of mitochondria and lysosomes may mutually intensify and exacerbate each other through metabolic stress-mediated signaling, contributing to the pathogenesis of AD^10^. Mitochondrial dysfunction induced by genetic mutations or Aβ and tau-mediated toxicity in AD pathogenesis associated with metabolic stress represses AMPK and transcription factor EB (TFEB), a master regulator of lysosomal genes, and triggers lysosomal hypoacidification, which induces the loss of lysosomal hydrolysis^10^. This mechanism may inhibit the degradation of damaged mitochondria by autophagy during mitochondrial dysfunction. Reciprocally, impaired lysosomal hydrolase is one of the many ways in which mitochondrial gene mutations are linked to ELN dysfunction. Therefore, ELN dysfunction can decrease mitophagy and increase the proportion of malfunctioning mitochondria. Furthermore, Aβ oligomers have been demonstrated to impede mitochondrial activation via mammalian target of rapamycin complex 1 (mTORC1)-dependent lysosomal amino acid sensing^10^. Taken together, the evidence suggests that in addition to individual mitochondrial and ELN dysfunctions, the crosstalk between mitochondria and ELN plays a crucial role in the pathogenesis of AD associated with metabolic syndrome. Additionally, a deeper investigation into the regulatory mechanisms that govern mitochondria–ELN crosstalk could provide novel strategies for mitigating AD progression in patients with metabolic syndrome.

## Microbial dysbiosis in metabolic diseases

Dysbiosis in the GMB is found in both metabolic disease and AD, and some of these changes are similar^11,13^, indicating that changes in the GMB in metabolic diseases may impact the development of AD. Therefore, identifying and regulating gut dysbiosis in metabolic diseases can help us understand disease pathogenesis. The typical GMB in humans consists primarily of *Firmicutes*, *Proteus, Actinomycetes, Bacteroides*, and *Fusobacteria*, which are crucial to metabolic health, and their dysbiosis is involved in the pathogenesis of diseases such as diabetes and obesity^40^. Dysbiosis of the GMB in both diseases is mainly caused by changes in the gut metabolome due to excessive metabolism of sugar, saturated fat, and protein and disruption of gut barrier integrity^41,42^. In diabetes, the GMB is associated with an increase in the abundance of bacteria with proinflammatory potential, such as *Clostridium clostridioforme*, *Prevotella capricopri*, and *Bacteroides vulgatus*; a decrease in the abundance of *Akkermansia muciniphila*, which improves gut barrier function and regulates inflammation; and a loss of bacteria producing short-chain fatty acids (SCFAs). Changes in the composition of the GMB and its metabolites result in increased intestinal permeability, insulin resistance, systemic inflammation, dysregulation of glucose metabolism, and neuronal dysfunction^41,43,44^.

Obesity also causes changes in the GMB, especially an elevated ratio of *Firmicutes* to *Bacteroidetes* and a decrease in SCFA-producing bacteria, which is associated with a decreased conjugative capacity to transfer genetic material between bacteria and a decrease in superoxide reductase, leading to oxidative stress in the gut. The observed change in bacterial populations leads to better food energy utilization, which is associated with higher levels of adiposity and metabolic dysfunction^45^. Hansen et al. revealed that the GMB inhibits the expression of the long noncoding RNA *Snhg9*, resulting in increased lipid absorption and storage. Increased *Snhg9* expression in mice resulted in decreased FA absorption, providing a defense against obesity generated by dietary factors and metabolic abnormalities^46^. Consequently, in metabolic diseases, dysbiosis in the GMB can affect gut function, systemic inflammation, and neurological function. Because GMB dysbiosis inherently induces changes in its metabolites, which may lead to the functional changes mentioned above, further exploration focusing on the function and regulatory mechanisms of metabolites is essential for expanding our knowledge of metabolic diseases and providing information for developing more effective AD treatment strategies.

## Pathophysiological role of GMB-derived metabolites and extracellular vesicles in AD

Several studies have revealed that GMB dysbiosis in AD and metabolic diseases results in abnormal production of microbial metabolites that exacerbate disease progression^12,42^. Notably, elucidating the specific targets of GMB-derived metabolites and their mechanisms of action could provide crucial insights into potential therapeutic strategies for restoring the impaired function of intracellular organelles such as mitochondria and endolysosomes, which play pivotal roles in the pathogenesis of metabolic disorder-associated AD. Recent studies have identified several key mitochondrial targets, such as adenine nucleotide translocator (ANT), voltage-dependent anion channel (VDAC), mitochondrial permeability transition pore (mPTP), and mitochondrial respiratory chain complexes, which interact with Aβ and contribute to imbalances in mitochondrial dynamics, biogenesis, and mitophagy, as well as impairments in mitochondrial energy metabolism, membrane potential and Ca^2+^ homeostasis, advancing AD pathogenesis^47–52^. In addition to mitochondrial targets, endolysosomal dysfunction has been linked to the disruption of vacuolar ATPase (V-ATPase) activity, a decrease in neuronal FKBP4/FKBP52, and inhibition of IST1 (IST1 factor associated with ESCRT-III) expression, which mediates the proteopathy and impairs autophagosome–lysosome fusion^53–55^. By targeting these specific pathways and identifying potential GMB-derived metabolites, we may be able to develop novel interventions that effectively reduce the detrimental effects of metabolic dysfunction on the onset and progression of AD. Therefore, understanding the relationships between changes in metabolic disease-specific microbial metabolites and disease phenotypes is important for the development of drugs targeting GMB-derived metabolites. Metabolites that originate in the gut and hormones transported through the vagus nerve can influence the phenotypes of neurons and glia within the central nervous system and BBB. This influence can subsequently affect processes such as oxidative stress, neuroinflammation, amyloidosis, tauopathy, and the overall development of AD^12^. In the pathogenesis of obesity and T2DM, metabolites originating from the GMB can contribute to the onset of insulin resistance, potentially triggering an inflammatory response^42^.

**Fig. 2:**
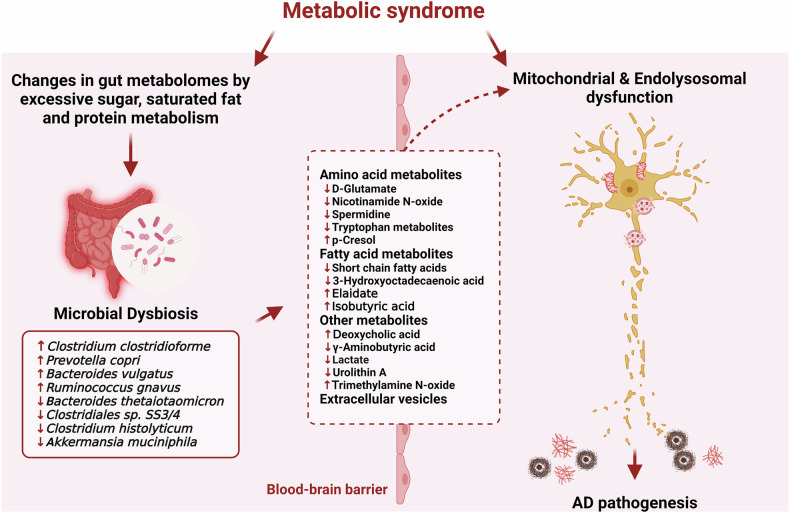
Potential role of GMB-derived metabolites and EVs in metabolic syndrome-associated AD pathogenesis. The pathogenesis of AD is attributed to mitochondrial and ELN dysfunction, which is caused by metabolic syndrome. Furthermore, a substantial amount of research is currently being conducted to explore the possibility that microbial dysbiosis might directly initiate these dysfunctions via GMB metabolites. Metabolic syndrome causes changes in the composition of the microbiota, resulting in shifts in the gut microbiota characterized by a reduction in beneficial bacteria and an increase in harmful bacteria (listed in the left text box). This shift leads to alterations in the metabolites and EVs generated by microbes (listed in the center text box), which might have functional variations based on the sources (such as amino acids, fatty acids, or others) of the produced metabolites. As a result, these metabolites can cross the BBB and be involved in inducing mitochondrial and ELN dysfunction in neurons. Created with BioRender.com.

GMB-derived metabolites transported into the bloodstream to the kidney can induce changes in mitochondrial function or interfere with mitophagy, which in turn promotes the progression of diabetic nephropathy^56^. Furthermore, GMB metabolites associated with metabolic diseases are pivotal in influencing GBA and the occurrence of psychiatric disorders^40^. As described above, mitochondrial dysfunction and ELN dysfunction are critical for the development of metabolic syndrome-associated AD, which may provide insight into the pathogenesis of AD induced by GMB metabolites. In the following section, we will categorize GMB-derived substances altered in metabolic diseases into amino acids, fatty acids, other metabolites, and extracellular vesicles (EVs), as these classifications can give us a subsequent hypothesis of how each metabolite regulates AD pathogenesis through specific signaling mechanisms associated with mitochondria and ELN function (Fig. 2). Consequently, we explored this issue by identifying metabolite-specific neuronal regulatory effects and mechanisms while simultaneously describing their therapeutic potential for AD. With an understanding of these connections, a more comprehensive approach to treating AD with targeted strategies can be developed.

### Amino acid metabolites

Microbial communities produce several amino acid metabolites with unique significance to human health and pathology. In patients with diabetes and obesity, altered GMB-derived tryptophan metabolites are significantly correlated with disease progression^40^. Tryptophan, an essential amino acid, serves as a precursor for serotonin, melatonin, niacinamide, and vitamin B3, among other vital compounds, through the three distinct indole, serotonin, and kynurenine pathways. Research conducted in clinical settings revealed that obese and diabetic individuals exhibited significant decreases in indole metabolites such as indole-3-acid-acetic acid (IAA), indole-3-propionic acid (IPA), and indole-3-lactic acid (ILA), a phenomenon linked to gut dysbiosis with *Bacteroides*, *Clostridium*, *Bifidobacterium*, and *Lactobacillus*^40^. In obese subjects, IAA, ILA, and IPA levels showed a negative relationship with serotonin and kynurenine/tryptophan levels, indicating a reduction in the microbial tryptophan metabolism pathways and an increase in the kynurenine and serotonin pathways^40^. Indole derivatives potentially interact with proteins involved in AD pathogenesis. After absorption from the intestines, IPA transits to the brain, where it neutralizes hydroxyl radicals, decreases DNA damage, alleviates neuroinflammation by inhibiting astrocytes and microglia and hinders the formation of Aβ fibrils^57^. Additionally, serotonin and melatonin, which are derived from tryptophan through the serotonergic pathway and are reduced in patients with AD, play a protective role in AD by increasing brain-derived neurotrophic factor expression in the hippocampus and prefrontal cortex and reducing intraneuronal Aβ accumulation and hippocampal apoptosis^15^. Serotonin is produced in the brain and the GMB, but it cannot cross the BBB. Therefore, the intermediate 5-hydroxytryptophan produced by GMB is important for the replenishment of serotonin in the brain. Furthermore, another aspect that is much discussed in connection with AD research relates to the kynurenine pathway, which produces both neuroprotective and neurotoxic metabolites. Kynurenic acid is an effective antagonist of the N-methyl-d-aspartate receptor (NMDA), while 3-hydroxykynurenine and quinolinic acid induce oxidative stress and neuroinflammation^15^. Another kynurenine pathway-derived gut metabolite, nicotinamide adenine dinucleotide (NAD^+^), plays a crucial role in many biological processes, and NAD^+^ and its precursors, such as nicotinamide (NAM), mononucleotide (NMN), and nicotinamide riboside (NR), are being investigated for their potential protective effects against neurodegenerative disorders and metabolic diseases^58^. Feng Li et al. explored the relationship between NAD-modified metabolites and mitochondrial function, particularly in the context of AD. Their findings revealed that a specific GMB metabolite, nicotinamide N-oxide (NAMO), which is produced by bacteria such as *Lactobacillus gasseri* and *Lactobacillus reuteri*, prevents mitochondrial dysfunction and neuroinflammation^59^. NAMO promotes NAD^+^-dependent mitophagy, effectively regulating its activity levels, and halts the progression of AD. Previous investigations have indicated a strong association between NAD^+^ and GMB, with a focus on the essentiality of the microbiota in NR metabolism. Furthermore, NR supplementation has been shown to normalize disturbed GMB compositions^60^. However, studies investigating the effect of NAD^+^ on AD have failed to identify any specific gut bacteria involved in this process that directly regulates NAD^+^ metabolism. NAD^+^ plays a critical role in regulating mitochondrial function and lysosomal acidification, highlighting its importance in cellular metabolism and health^61^. These findings underscore the multifaceted roles played by tryptophan metabolites in AD^62^.

The importance of tyrosine derivatives in relation to metabolic diseases and neurological disorders cannot be understated. Our knowledge of the effects of tryptophan metabolites must be improved. Although the host does not synthesize p-cresol, L-tyrosine is converted to p-cresol by some bacterial species, including *Fusobacteriaceae*, *Enterobacteriaceae*, *Clostridium difficile*, and *Coriobacteriaceae*, while bacteria such as *Escherichia coli* and certain *Clostridium* species contribute to phenol production^63^. In the analysis of metabolome features, individuals with diabetes and obesity have a p-cresol enrichment pattern, which modifies mitochondrial oxidative metabolism and increases anion superoxide production^64^. Atypical social conduct triggered by p-cresol has been linked to a reduction in the functioning of central dopamine neurons, which play a role in the social reward pathway^63^. Another study showed that p-cresol altered the expression of NMDAR subunits within the nucleus accumbens and hippocampus in both healthy and epilepsy-prone rats. In addition, the activity of Rac1, a member of the Rho GTPase family that regulates neuronal structure, was abnormally increased by p-cresol, whereas CREB phosphorylation was defective in the hippocampus^65^. Therefore, it is likely that the increase in GMB-derived tyrosine metabolites in patients with diabetes and obesity contributes to neurological disorders through various mechanisms that need to be further investigated.

The natural polyamine spermidine can be acquired through oral intake from external dietary sources or through production by commensal GMB, including *Bacteroides* and *Fusobacteria*, and through cellular biosynthesis. Research has shown that increased dietary intake of spermidine is linked to neuroprotection and improved insulin resistance, which alleviates the progression of obesity, diabetes, and neurological disorders^66^. Although GMB-derived spermidine has not been reported to be involved in AD pathology, the therapeutic effects of spermidine are mainly related to maintaining mitochondrial function through the enhancement of mitophagy and mitochondrial respiration, anti-inflammatory effects, and the induction of autophagy associated with ELN function^66^. Consequently, these mechanisms are likely to be involved in reducing neuroinflammation and soluble Aβ levels in the AD mouse model induced by spermidine^67^.

Another amino acid metabolite, D-glutamate, can be produced by gut bacteria containing glutamate racemases, such as *Corynebacterium glutamicum*, *Brevibacterium lactofermentum*, and *Brevibacterium avium*. These bacteria are reduced in AD patients^68^. Because D-glutamate acts as an agonist of the NMDA receptor and its precursor glutamine regulates the function of ELN through TFEB regulation^69^, it has been the potential to improve cognition in AD^68^. Furthermore, since reduced fecal and plasma glutamate levels have been positively correlated with reduced glutamate-producing bacteria in obese patients^70^, obesity-reduced D-glutamate may contribute to AD development. Together, these GMB-derived amino acid metabolites play a distinctive role in AD progression in metabolic diseases. The pathophysiological mechanisms of amino acid metabolites remain unclear, but given that some metabolites act by modulating mitochondrial and ELN functions, it is likely that others also modulate these functions. Furthermore, further research to elucidate the detailed regulatory mechanisms of amino acid metabolites on mitochondria and ELN function could contribute to the development of specific therapeutic strategies for AD.

### Fatty acid metabolites

SCFAs, such as butyrate, propionate, and acetate, are widely researched and prevalent metabolites produced by the GMB, including *Bifidobacteriaceae*, *Lachnospiraceae*, *Ruminococcaceae*, and *Lachnospiracea*^41,71^. Once absorbed by colon cells, SCFAs can be used as an energy source in the colonic mucosa, can enter the bloodstream, or can pass through the BBB to affect brain function. They can attach to G protein-coupled receptors found in enteroendocrine L cells and trigger the release of glucagon-like peptide 1 (GLP-1) and peptide YY (PYY), leading to increased energy expenditure, decreased food consumption, and improved regulation of glucose metabolism and insulin production^41^. Therefore, dysbiosis in bacteria and changes in SCFA concentration have been observed in patients with diabetes and obesity^40^. In fact, the presence of bacteria such as *Eubacterium ventriosum* and *Roseburia intestinalis* and an increase in acetate levels are linked to obesity, while bacteria such as *Oscillospira* spp., which produce butyrate, may be associated with being lean. In individuals with diabetes, there are reductions in the GMB responsible for the production of butyrate and butyrate plasma concentration^41^. Furthermore, the importance of SCFAs in AD has been highlighted by the observation of a reduced number of SCFA-producing bacteria in fecal samples from AD patients^12^.

SCFAs strongly inhibit histone deacetylases (HDACs), leading to epigenetic modifications in gene expression. This mechanism may attenuate the progression of AD by influencing oxidative stress, nutrient metabolism, and inflammatory processes. Previous research has indicated that propionate reduces damage to the mitochondrial structure, while butyrate enhances neuronal mitochondrial function by increasing OXPHOS metabolism and inhibiting oxidative stress through epigenetic regulation^72,73^. Moreover, butyrate administration through the inhibition of HDACs resulted in the restoration of memory function and the upregulation of genes associated with associative learning in the APP/PS1 mouse model of AD^74^. Given what we have previously reported and our previous research indicating that butyrate inhibits high cholesterol-induced neuronal amyloidogenesis^72^, reducing SCFAs in individuals with diabetes and obesity may offer a viable approach to boosting mitochondrial function, which may have therapeutic benefits for the treatment of AD. Furthermore, ELN may also be involved in SCFA-mediated disease-regulatory mechanisms, as we recently demonstrated that butyrate ameliorates diabetes-related cognitive impairment by improving Parkin-mediated mitophagy^14^. Finally, future studies investigating the effects and mechanisms of SCFAs on AD pathogenesis should be designed to distinguish between SCFAs because increased gut butyrate production is associated with improved insulin resistance while increased propionate production or absorption is associated with an increased likelihood of developing AD^75^.

Several studies have examined the distinctive functions of long-chain fatty acids (LCFAs) generated by gut bacteria. The first study indicated that the overproduction of certain FAs by gut bacteria, especially *Fusimonas intestini*, leads to intensified, exacerbated obesity. This is attributed to the generation of long-chain FAs such as elaidate, which impair intestinal epithelial integrity and promote metabolic endotoxemia^76^. In contrast, the second study highlighted the anti-inflammatory properties of a specific LCFA, 3-hydroxyoctadecaenoic acid, which is produced by *Escherichia coli Nissle 1917* and *Holdemanella biformis* and is beneficial for reducing symptoms of colitis. FA functions by activating peroxisome proliferator-activated receptor gamma (PPARγ) in epithelial cells and modulating inflammatory responses^77^. In summary, the contrasting yet significant roles of LCFAs, as exemplified by these studies, can have diverse effects ranging from anti-inflammatory effects to metabolic endotoxemia, highlighting the possible neuroprotective or neurotoxic effects of gut and brain signaling on the pathogenesis of AD.

Moreover, branched-chain fatty acids (BCFAs) have an impact on the pathogenesis of diabetes and obesity due to their effect on the gut environment as well as on general health; therefore, they should also be considered an important factor^78,79^. Metabolites formed by fermentation of chain amino acids can sometimes undergo branching by the addition of methyl groups and then elongation to reconstitute BCFAs by gut bacteria, including *Lactobacillus spp*. and *Bifidobacterium spp*.^80^. In obese mice, BCFAs, especially isobutyric acid and isovaleric acid, promote gluconeogenesis in hepatocytes and stimulate the mTORC1/S6K1 pathway^79^, which can inhibit autophagy and ELN function. Although there is no research on the effect of BCFAs on AD pathogenesis, these compounds may affect AD through the modulation of systemic inflammation, autophagy, and gut–brain signaling mechanisms. Overall, changes in GMB-derived fatty acid metabolites in metabolic diseases may affect the pathogenesis of AD. Although their effects and detailed molecular mechanisms need to be investigated further, their roles in mitochondrial and ELN dysfunction may be involved in the development of neurodegenerative diseases, including AD.

### Other metabolites

Recent studies have shown that GMB-derived bile acids, which are secondary bile acids converted from primary bile acids, affect systemic health, including the immune system, and can play a role in metabolic disease^41^. Because secondary bile acids serve as important ligands for Takeda G-protein-coupled receptor 5 (TGR5) and farnesoid X receptor, both of which play crucial roles in the regulation of glucose and lipid metabolism through glucagon-like pathogenesis peptide-1 (GLP1) release, dysbiosis of bile acid-producing bacteria and subsequent changes in the concentrations of secondary bile acids are associated with diabetes and obesity^40^. The levels of secondary bile acids such as ursodeoxycholate (UDCA), chenodeoxycholate (CDCA), and lithocholate (LCA) were reduced in obese mice and were associated with a reduction in GMB, *Clostridium sindens*, and serum GLP1 concentrations^81^, while the level of deoxycholic acid (DCA) increased, accompanied by an increase in *Lactococcus*, *Ruminococcus Coprococcus*, and *Blautia*. Notably, in AD individuals, compared with older adults with normal cognitive function, there were notable reductions in the serum levels of cholic acid, a primary bile acid, and elevations in secondary bile acids such as DCA, along with its glycine and taurine conjugated forms^82^. Because numerous bile acids and their receptors have been detected in the brain, bile acid-mediated signaling may affect AD pathogenesis^82^. The interaction between DCA and nicastrin, a subunit of gamma-secretase, was found to be responsible for Aβ accumulation^83^. Additionally, another secondary bile acid, tauroursodeoxycholic acid (TUDCA), inhibits Aβ accumulation and neuronal cell death and improves mitochondrial function by improving Pink1-mediated mitophagy^82^.

Gamma-aminobutyric acid (GABA), the primary inhibitory neurotransmitter, is predominantly synthesized and controlled by astrocytes and neurons. The tonic GABA current plays a crucial role in regulating various brain functions and cognitive processes, such as memory, learning, sensory perception, and circadian rhythms. Therefore, it is not surprising that not only are GABA levels reduced in the cerebrospinal fluid (CSF) of AD patients, but there is also a significant reduction in GABA in the neuronal components of the brain^84^. Interestingly, GMBs such as *Bacteroides*, *Bifidobacterium*, and *Lactobacillus* contribute to GABA production, as evidenced by studies showing that altered GMB can influence GABA levels^85^. Although direct studies focusing on the role of microbiota-derived GABA in AD are limited, existing research suggests its potential impact through the modulation of neuroinflammation and neural excitability. Furthermore, given that GABA administration improves insulin resistance in patients with high-fat-diet (HFD)-induced diabetes^86^, changes in GMB-derived GABA concentrations in metabolic diseases may affect AD pathogenesis.

Lactate is produced by certain species of gut microbiota, particularly those belonging to the *Lactobacillus* and *Bifidobacterium* genera, alleviating diabetes symptoms through the fermentation of carbohydrates^87,88^. Notably, lactate can be further metabolized by other gut bacteria to produce SCFAs^89,90^. Interestingly, a recent study demonstrated that lactate could modulate intracellular Mg^2+^ dynamics, leading to increased mitochondrial Mg^2+^ uptake from the ER and subsequent alterations in mitochondrial function^91^. Moreover, changes in CSF Mg^2+^ concentrations have been reported to be associated with AD, suggesting a potential link between Mg^2+^ homeostasis and AD pathogenesis^92^. A recent study reported that lactate-mediated lactylation of PIK3C3/VPS34, a key component of the ELN, via the acetyltransferase KAT5/TIP60^93^ facilitates the endolysosomal degradation pathway by enhancing its lipid kinase activity. This finding suggested that lactate can directly regulate ELN function through posttranslational modifications, in addition to its effects on mitochondrial Mg^2+^ homeostasis. Given these findings, GMB-derived lactate could exert similar effects on mitochondrial and ELN function and potentially cross the intestinal barrier, enter the circulation, and influence metabolic alterations associated with changes in the gut microbiota composition. However, further research is needed to directly investigate the impact of gut microbiota-derived lactate on mitochondrial and ELN function in the context of metabolic disorders and AD pathogenesis.

The gut microbiota interacts with diet and may also have an impact on health outcomes, many of which involve metabolites produced by the microbiota from dietary components that can impact the host. Urolithin A (UA) is a member of the urolithin family and is produced in the colon through the GMB-mediated conversion of the natural polyphenols ellagitannins and ellagic acid found in various berry species. Notably, the specific bacteria responsible for producing UA in the human gut remain unidentified, and only approximately 40% of the population have the natural ability to efficiently convert dietary precursors into UA^94^. However, recent studies have identified the positive effects of the direct administration of UA on health, aging, and age-related conditions. In diabetic and obese mice, UA administration attenuated triglyceride accumulation in the liver, reduced plasma levels of low-density lipoproteins, and improved systemic insulin sensitivity^94,95^. A reduction in both Aβ plaques and phosphorylated tau levels and subsequent enhancement of cognitive functions were observed after UA treatment in the APP/PS1 mouse model of AD^96^. Research has consistently demonstrated that UA improves mitophagy and improves mitochondrial function while also reducing exaggerated inflammation^94^. Additionally, given that UA maintains mitochondrial calcium homeostasis by modulating abnormal ER–mitochondria contact in metabolic syndrome^26^ and significantly induces autophagic flux in hippocampal neurons^97^, further research may shed light on detailed disease regulatory mechanisms, including mitochondrial and ELN functions.

Trimethylamine (TMA) is synthesized by intestinal microbes such as *Clostridia*, *Enterobacteriaceae*, *Klebsiella*, and *Citrobacter* from dietary phosphatidylcholine, lecithin, and L-carnitine, enters the portal circulation, and is oxidized in the liver to trimethylamine N-oxide (TMAO). Elevated TMAO concentrations and their effect on disease have mainly been investigated in metabolic syndrome-mediated atherosclerosis^41^, but a recent study suggested that elevated levels of plasma TMAO were correlated with a greater likelihood of developing newly diagnosed diabetes^98^, and an affirmative correlation was observed between elevated levels of circulating TMAO and obesity, as indicated by a greater body mass index^99^. A recent investigation detected TMAO in CSF, implying its penetration into the BBB and its potential relevance to neurological processes or conditions^100^. In particular, experimental findings in mice exposed to dietary TMAO revealed accelerated brain aging and cognitive decline^100^. Elevated levels of TMAO, which is linked to Aβ accumulation, tau aggregation, and synaptic damage through mitochondrial impairment and superoxide production within neurons and glia, have been observed in individuals with AD and aged mice^101^.

### Extracellular vesicles

Recent findings have elucidated the translocation and existence of gut microbiota-derived extracellular vesicles (GMEVs) in addition to GMB-derived metabolites^102^. GMEVs exhibit various sizes, typically ranging from 20 to 400 nm, and are identifiable in various body fluids, including urine, serum, and CSF, as well as feces and intestinal aspirates. They can contain diverse bioactive compounds, such as proteins and nucleic acids, which can be transported over varying distances to regulate significant biological processes, thereby influencing the overall health of the host organism. In the development of diabetes and obesity, GMEVs can alter the permeability of the gut barrier and induce intestinal inflammation, increasing insulin resistance and triggering inflammation^103^. In fact, metagenomic analyses revealed the existence of EVs of the *Proteobacteria* phylum in the serum of individuals with diabetes, which inhibits insulin-induced activation of the insulin receptor substrate 1 in HFD-fed mice^104^. Furthermore, the outer membrane vesicles (OMVs) produced by the gram-negative bacterium *Porphyromonas gingivalis* also decrease insulin-stimulated glycogen synthase kinase 3β signaling^102^. However, it is important to keep in mind that some bacterial EVs, such as OMVs derived from *Akkermansia muciniphila*, which reduce adipocyte size and increase fatty acid oxidation in obese mice^102,104^, have shown positive results in disease prevention. From this perspective, EVs derived from bacteria that have adverse effects on diabetes and obesity may also have negative effects on AD.

In the brain, EVs affect neurons and glia by delivering proteins, lipids, RNA, and DNA, and these vesicles may contain inflammatory mediators or signaling molecules that modulate AD pathogenesis^105^. However, there are some preliminary suggestions that certain substances in GMEVs might be involved in cognitive impairment and the pathogenesis of AD. It was previously observed that among young mice undergoing fecal transplantation, cognitive impairments were correlated with the presence of two bacteria, *Paenalcaligenes hominis* and *Escherichia coli*^106^. Interestingly, EVs from *Paenalcaligenes hominis* promoted cognitive dysfunction, whereas those from *Escherichia coli* did not. In addition, the gut–liver axis may play a role in neurological functional changes through microbiota-derived metabolites and EVs influencing liver function^107^. Furthermore, a recent study suggested that GMEVs not only serve as intercellular communicators but also inhibit Aβ peptide aggregation^108^. They can also regulate functions within the CNS through substances such as serotonin. Furthermore, they can be used as potential biomarkers due to their involvement in the modulation of diseases for the early detection of AD. Although direct research linking these GMEVs to AD has not yet been extensively conducted, the literature suggests a potential connection between metabolic disease-induced GMEVs and the pathogenesis of AD. Furthermore, considering that the inhibitory effect of *Proteus mirabilis*-derived OMVs on bone loss was established by mitochondrial-dependent apoptosis^109^, it is quite possible that GMEVs, which have not yet been fully characterized, affect the disease by modulating the function of mitochondria or ELNs in AD, and further studies are needed to understand the specific underlying mechanisms involved. This may open new avenues for therapeutic intervention and provide a deeper understanding of the pathogenesis of AD.

### Therapeutic strategies targeting the GMB–metabolite–mitochondria–ELN axis in AD

Emerging evidence indicates an intricate interaction between GMB-derived metabolites, metabolic syndrome, and cellular dysregulation, such as mitochondrial and ELN dysfunction, in AD pathogenesis (Table 1). To further understand these associations, it is important to investigate the detailed mechanisms by which alterations in metabolic syndrome-specific bacterial species and their metabolites occur and how altered GMB-derived metabolites modulate mitochondrial or ELN functions and mitochondria-ELN crosstalk. Furthermore, by integrating multiomics data platforms, which encompass microbial, metabolomic, transcriptomic, and proteomic datasets, analyzing these relationships from a systems biology perspective will be instrumental in revealing their complex interplay. Recent progress, such as the application of UPLC–MS/MS and deep learning models to detect biomarkers of metabolic syndrome and AD, demonstrates the ability of metabolomics and metagenomics to provide valuable insights for preventive and therapeutic approaches^110^. Additionally, in vitro cell models and different in vivo animal models, including organoid–microbe coculture systems, conventional, germ-free, advanced genetic models, and gnotobiotic mice, are necessary to validate the direct links between microbial factors, subcellular defects, and AD progression^111–113^.

**Table 1:** Potential mechanisms of microbial metabolites altered in metabolic syndromes contributing to AD development.

Category	Microbes	Metabolites	Mechanism of Action	Refs.
Amino acid metabolites	*Bacteroides, Clostridium, Bifidobacterium, Lactobacillus*	Indole-3-acetic acid, indole-3-propionic acid, indole-3-lactic acid	Neutralizes hydroxyl radicals, decreases DNA damage, and alleviates neuroinflammation via Aryl hydrocarbon receptor activation	^15,40,57^
	*Lactobacillus gasseri, Lactobacillus reuteri*	Nicotinamide n-Oxide	Prevents neuroinflammation and restores NAD^+^-dependent mitophagy.	^59^
	*Bacteroides, fusobacteria*	Spermidine	Improves mitophagy and mitochondrial respiration, induces autophagy-mediated ELN function, and inhibits inflammation, leading to reduction of Aβ levels via reduced NF-κB phosphorylation.	^66,67^
	*Corynebacterium glutamicum, Brevibacterium lactofermentum, Brevibacterium avium*	D-Glutamate	Acts as an agonist of the NMDA receptor, modulates TFEB-mediated ELN function through glutamine metabolism.	^68–70^
	*Fusobacteriaceae, Enterobacteriaceae, Coriobacteriaceae, Clostridium difficile, Escherichia coli*	p-Cresol, phenol	Alters mitochondrial oxidative metabolism and increases anion superoxide production. Changes the expression of NMDAR subunits within the nucleus accumbent and hippocampus and stimulate Rac1 activity.	^63–65^
Fatty acid metabolites	*Firmicutes, Oscillospira, Akkermansia muciniphila*	Butyrate	Maintains mitochondrial OXPHOS metabolism, inhibits oxidative stress, and improves mitophagy through HDAC inhibition-mediated epigenetic modification.	^14,72,74,75,126^
	*Bifidobacteria, Lactobacilli, Eubacterium ventriosum, Roseburia intestinalis*	Acetate	Improves mitochondrial respiratory chain function and suppresses oxidative stress by reducing H3K4me3- and H3K9ac-mediated metabolic gene repression, consequently modulating microglial phagocytosis and disease progression during neurodegeneration.	^75,126,127^
	*Bacteroidetes, Lachnospiraceae*	Propionate	Maintains mticohondrial structure and regulates inflammation and BBB permeability via increase of occludin, claudin-5 and ZO-1 localization and inhibition of NF-κB activation.	^73,75,126,128^
	*Fusimonas intestini*	Elaidate	Impairs intestinal epithelial integrity, promotes metabolic endotoxemia via reduction of ZO-1, occludin. JAM1 expression.	^76^
	*Escherichia coli Nissle 1917, Holdemanella biformis*	3-Hydroxyoctadecaenoic acid	Activates PPARγ-mediated anti-inflammatory responses.	^77^
	*Lactobacillus, Bifidobacterium*	Isobutyric acid, isovaleric acid	Modulates systemic inflammation through stimulation of mTORC1-inhibited autophagy.	^78–80^
Other metabolites	*Enterobacteriaceae, Escherichia coli, Klebsiella, Citrobacter*	Trimethylamine N-oxide	Impairs mitochondrial function, causes oxidative stress, and inhibits mTOR signaling within neurons and glia, leading to Aβ accumulation, tau aggregation, and neuroinflammation.	^98,99,101,129,130^
	*Lactobacillus, Bifidobacterium, Bacteroides*	γ-Aminobutyric acid	Modulates neuroinflammation and neural excitability with NMDAR/AMPAR expression ratio changes in postsynaptic neuron.	^84–86,131–133^
	*Lactococcus, Ruminococcus, Coprococcus,Blautia*	Deoxycholic acid	Interacts with nicastrin, which abnormally produces Aβ.	^81–83,134–137^
	*Gordonibacter, Ellagibacter*	Urolithin A	Inhibits Aβ accumulation and tau aggregation by enhancing mitophagy, mitochondrial function and autophagy flux, maintaining mitochondrial calcium homeostasis, and reducing neuroinflammation.	^26,94–97^
Extracellular vesicles	*Paenalcaligenes hominis, Escherichia coli*	Gut microbiota-derived extracellular vesicles, outer membrane vesicles	Impacts vagus nerve with cognitive dysfunction and inhibits Aβ aggregation.	^102,103,106,108^

On the therapeutic front, targeting the metabolic syndrome-altered GMB–metabolite–mitochondria–ELN axis could be a promising strategy for intervening in AD. This approach underscores the need to develop integrated and personalized treatment modalities that go beyond traditional single-target therapies. Several strategies have been proposed to modulate the GMB, including the use of prebiotics, probiotics, synbiotics, and postbiotics. Prebiotics are nondigestible food components that stimulate the growth and activity of advantageous gut bacteria, thus promoting a healthy gut microbiome. Probiotics involve the administration of beneficial bacteria to restore a healthy gut microbiome, which may involve reconstitution with a single bacterial treatment through nanoencapsulation techniques^114–116^. Synbiotics combine both probiotics and prebiotics to achieve a synergistic effect on the GMB. However, these approaches face challenges such as the survival and colonization of the introduced bacteria in the gut, which may limit their long-term efficacy. Given the anaerobic nature of many gut bacteria, postbiotics, bioactive compounds produced by microorganisms, may offer a promising alternative for modulating GMB-derived metabolites in the context of AD^117^. Postbiotics can be administered directly to exert beneficial effects without the need for live bacteria, thus overcoming potential challenges such as the survival and colonization of introduced bacteria in the gut. These postbiotic compounds include heat-killed fractions of probiotic bacteria and metabolites derived from GMB, such as lipoteichoic acid, butyrate, tryptophan metabolites, rhamnose-rich exopolysaccharides, and UDCA. These postbiotics may directly or indirectly regulate mitochondrial and endolysosomal functions through various mechanisms, including the modulation of autophagy, host metabolism, systemic immunity, and antioxidative effects, thereby potentially influencing the pathogenesis of metabolic syndrome-associated AD^118–125^. However, further research is required to identify the specific compounds and their mechanisms of action, as well as to determine the optimal dosing and delivery methods for the clinical application of postbiotics.

In addition to these targeted interventions, nutritional strategies aimed at modulating the GMB and its metabolites may play a crucial role in the prevention and management of AD. Accumulating evidence suggests that diet can significantly influence the composition and function of the GMB, with implications for metabolic health and brain function. Investigating how diet affects GMB–mitochondria–ELN communication in the management of metabolic syndrome may lead to the development of nutritional strategies for both AD prevention and treatment. These strategies may include dietary interventions, such as increasing the consumption of fiber-rich foods and limiting the intake of processed and high-fat foods, as well as lifestyle modifications, such as regular exercise and stress reduction.

## Conclusions and prospects

In summary, elucidating the relationship between GMB metabolites and subcellular processes in the pathogenesis of metabolic syndrome-associated AD has the potential to provide novel perspectives for its treatment. Exploring the therapeutic strategies that directly influence the functions of mitochondria and endolysosomes within the broader scope of GMB and metabolite research represents a promising frontier in AD therapy. However, the current lack of research directly linking GMB-derived metabolites to mitochondrial and endolysosomal impairment in AD pathogenesis highlights the necessity for comprehensive investigations. Recent innovations in metabolomics and metagenomics, along with the utilization of various in vitro and in vivo models, can help validate the direct links between GMB, subcellular defects, and AD progression. Targeting the GMB–metabolite–mitochondria–endolysosome axis altered by metabolic syndrome could emerge as a promising approach for alleviating AD pathology, emphasizing the need for integrated and personalized therapeutic approaches. Translating the insights achieved from studying the GMB–metabolite–mitochondria–endolysosome axis into clinical practice will require meticulous consideration of various factors, including individual variations in GMB composition, the safety of GMB-targeted interventions, and the establishment of noninvasive biomarkers. Overcoming these concerns will be pivotal in bridging the gap between preclinical investigation and clinical application, enabling the development of effective and safe interventions directed at the GMB–metabolite–mitochondria–endolysosome axis in AD. Future research should concentrate on validating the direct associations between specific metabolites and cellular dysfunction, developing personalized therapeutic approaches, and addressing the hurdles linked to translating these findings into clinical practice.
